# Decreased suicide rates in recent antidepressant clinical trials

**DOI:** 10.1007/s00213-018-4856-1

**Published:** 2018-02-26

**Authors:** Arif Khan, Kaysee Fahl Mar, Sagarika Gokul, Walter A. Brown

**Affiliations:** 1 Northwest Clinical Research Center, 1951 152nd Pl. NE Suite #200, Bellevue, WA 98007 USA; 2 Department of Psychiatry, Duke University School of Medicine, Durham, NC USA; 3 Department of Psychiatry and Human Behavior, Brown University, Providence, RI USA

**Keywords:** Suicide, Suicide attempt, Clinical trials, Antidepressants

## Abstract

**Rationale:**

The last systematic analysis of suicidality in antidepressant clinical trials submitted for approval by the US Food and Drug Administration was in 2000. Given the attention to suicide and antidepressants in the early 2000s, the authors aimed to evaluate if there have been any changes in suicide rates in antidepressant clinical trials following 2000.

**Objective and Methods:**

The Integrated Safety Summary data from approval packets for 14 investigational antidepressant programs (1991–2013, 40,857 patients, 10,890.5 exposure years) were used to calculate suicides and suicide attempts per 100,000 patient exposure years (standardized rates) for antidepressant and placebo treatment groups separately. Suicides/suicide attempt rates, mean age, and percent female were compared between 1991 and 1998 (pre-2000) and 2002–2013 (post-2000). Drug-placebo differences in suicide/suicide attempt rates were explored.

**Results:**

Among antidepressant-treated patients, the standardized suicide rate decreased significantly from pre- to post-2000 (643.6 to 25.8, *p* < 0.0001) as did the standardized suicide attempt rate (3975.7 to 645.4, *p* < 0.0001). For placebo-treated patients, the decrease was not significant for suicide rate (471.1 to 174.2, *p* = 0.66) but was significant for suicide attempt rate (from 3538.3 to 522.6, *p* < 0.001). Regression analysis showed a similar pattern with suicide/suicide attempt rates decreasing over time. None of the drug-placebo comparisons in suicide or suicide attempt rates were statistically significant. There was no change in percent female or mean age of patients in trials pre- and post-2000.

**Conclusions:**

Deaths by suicide and suicide attempts have decreased significantly in antidepressant clinical trials following 2000 compared to the decade before 2000. Basic demographic features of the patients have remained consistent and medication treatment effects on suicidality were not apparent. These findings may reflect enhanced screening procedures and effective exclusion of suicidal patients in clinical trials for depression.

## Introduction

Although concern about the induction of suicidal behavior with antidepressants has been present since antidepressants were discovered, it was in 1990 that Teicher et al. (1990) reported unexpected, new-emerging suicidal ideation and obsession in depressed patients treated with fluoxetine. As the authors noted, this finding was counterintuitive; people who died by suicide are known to have lowered serotonin activity in the brain and serotonin re-uptake inhibitors (SSRIs) such as fluoxetine are believed to act by increasing available serotonin.

Following this serendipitous discovery, considerable research (Bridge et al. 2007; Gibbons et al. 2012; Olfson et al. 2006; Tiihonen et al. 2006) has aimed to assess suicide risk with SSRIs and other antidepressants. Data from antidepressant clinical trials in adults conducted between 1991 and 2000 (Khan et al. 2000) demonstrated that the rates of death by suicide were low overall and no significant differences were seen in suicide rates between antidepressant and placebo-assigned patients. Despite this, there has been ongoing concern about including suicidal patients in clinical trials of antidepressants. As a response to this concern, not only has there been an increase in suicide research but also attempts to control the rate of suicide in these trials have intensified in the recent decade.

As can be reported from experience in clinical trial investigation, inclusion/exclusion criteria have become more stringent in the past decade and a half, particularly regarding the inclusion of those at apparent risk of suicide. As part of the effort to address the rate of suicide in antidepressant trials, the practice of excluding potentially suicidal patients from these trials has become more tightly regulated by the US Food and Drug Administration (FDA). This effort has historically included use of clinical impressions of suicide risk and items on measurement scales that evaluate suicidality as a symptom (such as item-10 on the Montgomery-Asberg Depression Rating Scale). Following the intensified interest in the rate of suicide in antidepressant trials around 2000, operationalized procedures focused on suicide risk assessment, such as the Columbia-Suicide Severity Rating Scale (C-SSRS) (Posner et al. 2011) and the Sheehan Suicidality Tracking Scale (S-STS) (Coric et al. 2009), were developed and such tools were implemented by sponsors for use in screening out suicidal patients from clinical trials. Although known among investigators and staff working on clinical trials, such changes in the recent decade regarding the screening tools or selection criteria for clinical trials of Major Depressive Disorder (MDD) have not been quantified due to a lack of access to such proprietary protocol information.

Since 2000, there have been seven new antidepressants approved by the US Food and Drug Administration, offering a substantial amount of data regarding suicides in recent antidepressant clinical trials. In light of the increase in attention to suicide in antidepressant clinical programs following 2000 and because there has not been a systematic review of the suicide rates in these more recent trials, we aimed to evaluate the prevalence of death by suicide and of suicide attempts in programs over the past 17 years compared to those prior to 2000.

Given the heightened attention to and enhanced screening for apparent suicide risk following 2000, we hypothesized that deaths by suicide and suicide attempts in antidepressant programs would have decreased after 2000 compared to programs approved prior to 2000. To test this assumption, we evaluated data from the Integrated Safety Summaries included in the New Drug Approval registration packets for 14 antidepressants approved 1991–2013. In order to test for a potential change following 2000, we compared rates of suicide and suicide attempts per 100,000 patient exposure years (PEY) in antidepressant and placebo treatment groups separately between pre-2000 antidepressant programs and post-2000 programs. Additionally, we examined any changes in suicide and suicide attempt rates for antidepressant and placebo over time as a continuous measure by using a weighted regression model. Differences in suicide and suicide attempt rates were compared between antidepressant and placebo treatment to explore any potential treatment effects. Finally, it is important to consider the population samples and their characteristics, particularly with regard to known modifiers of suicide rates like age and sex. Notably, other investigators (Stone et al. 2009) in a very large database of placebo-controlled trials found an age dependent relationship between suicidal behaviors and the use of all antidepressants. We examined if there were any changes to the reported demographic features of enrolled patients following 2000 that may influence the observed suicide rates.

## Methods and materials

### FDA database

We used the US FDA database (http://www.accessdata.fda.gov/) (FDA ACCESS DATA 2016) because it contains standardized and verified reports of safety data from antidepressant clinical trials. Reviewers for New Drug Approval (NDA) programs assess the quality of safety data recorded by sponsors seeking FDA approval for an investigational antidepressant. Reports of these safety data are collected into the Integrated Safety Summaries (ISS), which are presented in the reviews submitted to the FDA for approval. All data for this analysis was recorded from safety summaries submitted to the US FDA which is inclusive of both domestic (US-only) trials and international trials registered with the FDA. Because these data are public domain and void of identifying qualities, an ethical approval was not needed for this study.

### Selection of programs

For this analysis, we included programs for investigational antidepressants or new molecular formulations (such as extended-release formulations) of antidepressants indicated for the treatment of major depressive disorder (MDD) in adults (> 18 years old).

Safety datasets for two programs that met inclusion criteria, fluoxetine (1987) and bupropion (1996), were not evaluable in the context of this analysis due to absence of data necessary to calculate either standardized suicide or suicide attempt rates per 100,000 patient exposure years. Therefore, there were 14 antidepressant approval programs included in this analysis.

These 14 antidepressant programs were categorized into two groups by year of approval, resulting in 7 programs in the pre-2000 group: sertraline hydrochloride (1991), paroxetine hydrochloride (1992), venlafaxine hydrochloride (1993), nefazodone hydrochloride (1994), mirtazapine (1996), venlafaxine hydrochloride ER (1997), and citalopram (1998). Likewise, there were 7 programs in the post-2000 group: escitalopram oxalate (2002), duloxetine hydrochloride (2002), desvenlafaxine succinate (2008), trazodone hydrochloride ER (2010), vilazodone hydrochloride (2011), levomilnacipran hydrochloride (2013), and vortioxetine hydrobromide (2013).

### Integrated Safety Summaries

As part of the submission of a New Drug Approval program, the Integrated Safety Summaries report adverse events that occurred in Phase II and Phase III trials and typically 30 days after treatment conclusion. These safety summaries included data from patients in randomized placebo and active-controlled acute trials as well as open-label and ongoing safety studies. This collection of information constitutes the original safety population dataset. Many, but not all programs have published safety updates in addenda to the original dataset. As a way to uniformly collect safety data from these programs, we selected the original ISS as it was presented at the time of approval from the FDA in all cases where it was available and reported suicides.

Where there were discrepancies in reporting or substantial differences in the selection of safety data from program to program, we applied certain inclusion/exclusion criteria to increase comparability between programs. We only included data from safety populations of patients with MDD, completed studies, and those in which PEY was clearly reported or an adequate mean duration of exposure was given to calculate PEY for at least one of our suicide measures. In cases where suicides were reported outside of the treatment timeframe (during screening or > 30 days following treatment discontinuation), we excluded these data points from our analysis. The original ISS reports met these criteria for 11 out of the 14 programs we evaluated.

However, in the case of nefazodone (1994), the original ISS did not provide data regarding suicides, and so we used the updated version of the ISS, which included original and added safety data gathered from studies completed post-approval. Additionally, trazodone ER (2010) reported only one Phase III trial for safety consideration, therefore this trial sample was regarded as the safety population for this program. Finally, the ISS for vortioxetine (2013) included grouped estimations of PEY for short-term and long-term studies, but did not control for overlap between patients who participated in both by rolling over. Therefore, in this case, we only included the data from short-term studies because we could verify that this population represented unique rather than redundant data entries.

### Definition of terms

Death by suicide: the raw number of completed and confirmed deaths by suicide was reported for each program and treatment assignment. These deaths were judged by a medical examiner to be caused by suicide.

Suicide attempts: we were able to record the number of suicide attempts for those ISS reports that tabulated suicidal attempts as a separate category of adverse events. Understanding that classification of adverse events as suicide attempts is dependent on the criteria used by reviewers, we aimed to evaluate the safety data as it is presented in the FDA reviews. For this reason, we used data from any report of “suicide attempts” as defined by the medical examiners within that program’s safety review.

Patient exposure years (PEY): sum of the duration of exposure to the assigned treatment condition from each patient in the safety population. PEY was taken as reported in the safety summaries and verified as corresponding to the population in which the suicide deaths and suicide attempts occurred. In cases where PEY was not calculated in the review, sufficient data to calculate an estimate of the exposure were given. This allowed for calculation of PEY based on the given mean duration of exposure multiplied by the number of patients.

Suicides and suicide attempts per 100,000 PEY: as a standardized measure to compare suicide rate while controlling for number of patients and the duration of their exposure, we calculated suicide and suicide attempts per 100,000 patient exposure years. This is calculated by dividing 100,000 by the given PEY for the program treatment condition and multiplying this by the number of suicides or suicide attempts. The resulting number reflects the proportional suicide rate while allowing for simplified comparisons between programs with varying PEY for their data.

### Statistical measures

Statistical measures were generated with IBM Statistical Package for the Social Sciences (SPSS). Fisher’s Exact tests (2-sided) were used to compare groups (pre-2000 and post-2000) using Poisson statistics on measures of suicide and suicide attempt rate per 100,000 PEY. As a way to test for rate changes over time as a continuous measure, simple PEY-weighted (as a measure of precision) regressions were used to measure the significance of the relationship between the year of approval and deaths by suicide in Fig. 3. Independent sample *t* tests were used to compare demographic data (mean age and percent female) of patient populations pre- and post-2000.

## Results

### Summary of program data

Table 1 provides a summary of all included programs and their corresponding safety population and suicide data by treatment condition (antidepressant or placebo). Two programs, venlafaxine (1993) and venlafaxine ER (1997), did not report data regarding suicide attempts. Therefore, five programs (5/7, 71%) were used to calculate the suicide attempt rate pre-2000. Additionally, patient exposure years were not given for placebo treatment in two post-2000 programs, duloxetine (2002) and levomilnacipran (2013). However, the reported number of suicides and suicide attempts for placebo in these two programs was zero, allowing for the calculation of suicides and suicide attempts per 100,000 PEY.

**Table 1:** Summary of suicide data from the Integrated Safety Summaries (ISS) of 14 investigational antidepressants including N patients, patient exposure years (PEY), N suicides, and N suicide attempts

Approval year	Program	Antidepressant		Placebo		N suicides		N suicide attempts	
		N	PEY	N	PEY	AD	Placebo	AD	Placebo
1991	Sertraline	2053	507.9	786	209	2	0	9	5
1992	Paroxetine	2963	1008	554	72	5	2	40	6
1993	Venlafaxine	2181	879	451	100	3	1	--	--
1994	Nefazodone	3496	1018	875	204	9	0	12	1
1996	Mirtazapine	2425	671.7	494	71.4	8	0	29	3
1997	Venlafaxine ER	705	161.6	285	42.4	1	0	--	--
1998	Citalopram	4168	1347.7	691	150.3	8	1	91	10
1991–1998 totals		17,991	5593.9	4136	849.1	36	4	181	25
2002	Escitalopram	715	99	592	83	0	0	4	0
2002	Duloxetine	2314	754	723	**--**	0	0	7	0
2008	Desvenlafaxine	2667	1137.2	803	112.8	1	0	4	1
2010	Trazodone ER	202	25.9	204	28.8	0	0	0	1
2011	Vilazodone	2177	551.5	997	136.1	0	1^**a**^	3	1
2013	Levomilnacipran	2655	899.5	--	--	0	0	4	0
2013	Vortioxetine	3060	406.3	1621	213.4	0	0	3	0
2002–2013 totals		13,790	3873.4	4940	574.1	1	1	25	3

**--** indicates that data was not available

^a^Event occurred prior to first dose of treatment

### Suicides per 100,000 PEY (Fig. 1)

Antidepressant-exposed patients in programs pre-2000 had 24.9 (CI 4.8–511.5, *p* < 0.0001) times the rate of deaths by suicide (per 100,000 PEY) as compared to antidepressant-exposed patients in programs post-2000. The decrease in antidepressant treatment deaths by suicide (per 100,000 PEY) from 643.5 (CI 450.7–890.9) in programs 1991–1998 to 25.8 (CI 0.7–143.8) in programs 2002–2013 was statistically significant according to Fisher’s Exact test (*z* = 4.73, *p* < 0.0001).

**Fig. 1:**
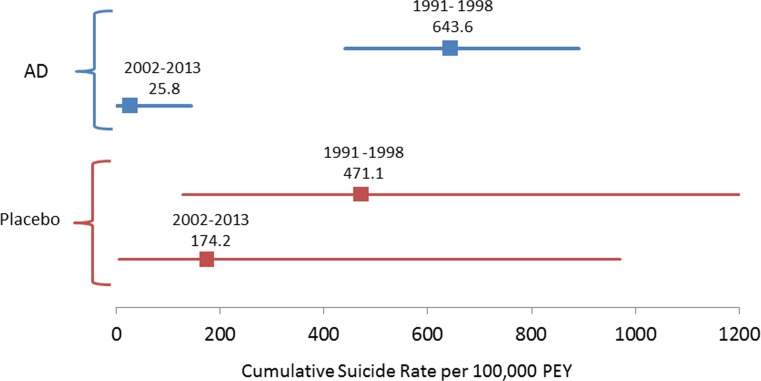
Forest plot of the cumulative suicide rate per 100,000 patient exposure years for programs between 1991 and 1998 and 2002–2013 separated by antidepressant (AD) and placebo treatment conditions

Placebo-exposed patients in antidepressant programs pre-2000 did not have a significantly different rate of deaths by suicide (per 100,000 PEY) as compared to placebo-exposed patients in programs post-2000. The decrease in placebo treatment deaths by suicide (per 100,000 PEY) from 471.1 (CI 128.4–1206.2) in programs 1991–1998 to 174.2 (CI 4.4–970.5) in programs 2002–2013 was not statistically significant according to Fisher’s Exact test (*z* = 0.93, *p* = 0.66).

### Suicide attempts per 100,000 PEY (Fig. 2)

Antidepressant-exposed patients in antidepressant programs pre-2000 had 6.16 (CI 4.11–9.54, *p* < 0.0001) times the suicide attempts (per 100,000 PEY) as compared to antidepressant-exposed patients in programs post-2000. The decrease in antidepressant treatment suicide attempts (per 100,000 PEY) from 3975.7 (CI 3417–4598) in programs 1991–1998 to 645.4 (CI 417.6–952.8) in programs 2002–2013 was statistically significant according to Fisher’s Exact test (*z* = 9.74, *p* < 0.0001).

**Fig. 2:**
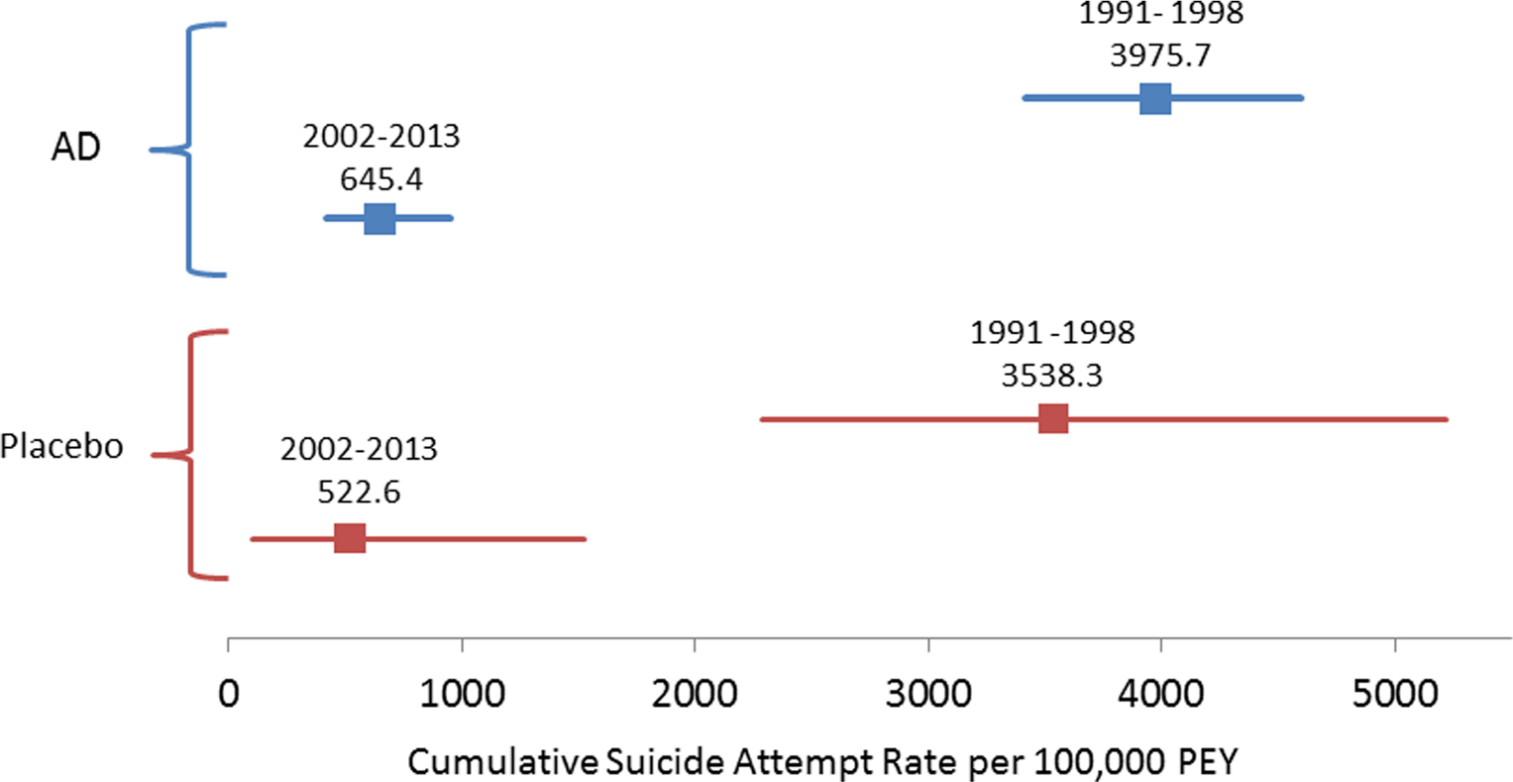
Forest plot of the cumulative suicide attempt rate per 100,000 patient exposure years for programs between 1991 and 1998 and 2002–2013 separated by antidepressant (AD) and placebo treatment conditions

Placebo-exposed patients in antidepressant programs pre-2000 had 6.77 (CI 2.26–28.21, *p* < 0.001) times the suicide attempts (per 100,000 PEY) as compared to placebo-exposed patients in programs post-2000. The decrease in placebo treatment suicide attempts (per 100,000 PEY) from 3538.3 (CI 2289–5222) in programs 1991–1998 to 522.6.4 (CI 105–1527) in programs 2002–2013 was statistically significant according to Fisher’s Exact test (*z* = 3.6, *p* < 0.001).

### Deaths by suicide and suicide attempts over time (Figs. 3 and 4)

When plotted using regression (weighted by patient exposure years), the rate of death by suicide per 100,000 PEY decreased significantly over time in the antidepressant treatment group (*β* = − 34.0, *R*^2^ = 0.49, *p* < 0.001). The decrease in the rate of deaths by suicide in placebo-treated patients was not significant (*p* = 0.22).

**Fig. 3:**
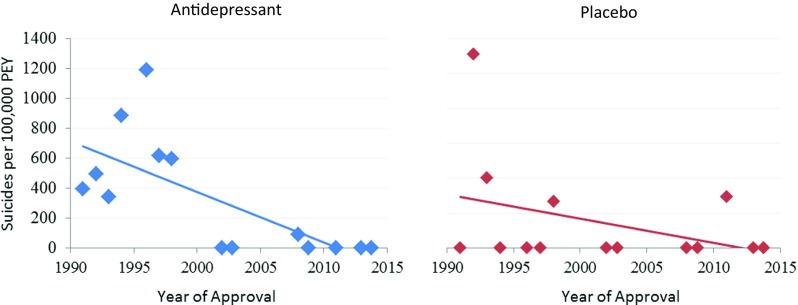
Rates of suicide per 100,000 patient exposure years plotted with year of approval. Rates from antidepressant (blue) and placebo (red) are plotted separately

**Fig. 4:**
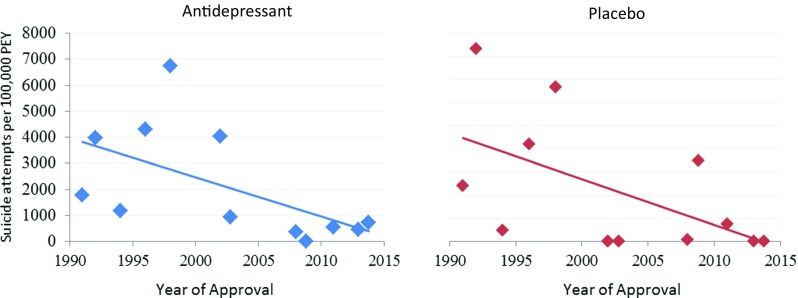
Rates of suicide attempts per 100,000 patient exposure years plotted with year of approval. Rates from antidepressant (blue) and placebo (red) are plotted separately

Significance was also found for the decrease over time in the rate of suicide attempts per 100,000 PEY in the antidepressant treatment group (*β* = − 150.3, *R*^2^ = 0.33, *p* = 0.016) and was also found for the decrease in suicide attempt rate for placebo treatment (*β* = − 196.3, *R*^2^ = 0.29, *p* = 0.043).

### Drug-placebo differences in suicide and suicide attempt rates

There were no statistically significant comparisons between antidepressant and placebo treatment exposure in deaths by suicide or suicide attempts per 100,000 PEY. In pre-2000 programs, the Fisher’s Exact comparison between antidepressant and placebo groups was not significant for death by suicides (*p* = 0.65) or suicide attempts (*p* = 0.60). Post-2000 programs had similar findings with insignificant differences found between treatments in deaths by suicide (*p* = 0.24) and suicide attempts (*p* = 0.5).

### Demographics of patients over time

There was no evidence of a change in patient demographics in clinical trials. The mean age for patients in programs pre-2000 was 45.2 (± 3.0) and for post-2000 it was 42.9 (± 1.7) (*t* = 1.63, *p* = 0.142). Additionally, there was no evident change in the percentage of female patients with 59.7% (± 13.7) female in programs pre-2000 and 63.0% (± 3.8) female post-2000 (*t* = − 0.56, *p* = 0.60).

### Sensitivity analysis

We conducted a post-hoc sensitivity analysis as a way of examining if including the program for nefazodone, with its limited safety dataset, affected the results of this analysis significantly. The results of this analysis revealed that excluding the data from nefazodone did not result in significant differences in the death by suicide or the suicide attempt rate per 100,000 PEY and that the direction and significance of group comparisons between pre-2000 and post-2000 were not substantially altered.

## Discussion

The aim of this study was to evaluate the prevalence of death by suicide and suicide attempts in antidepressant and placebo treatment groups of antidepressant clinical trials over the past 17 years and to compare these rates to those from programs conducted prior to 2000. Deaths by suicide in antidepressant-treated patients and suicide attempt rates in both antidepressant and placebo-treated patients decreased significantly in more recent programs. Overall, these data support our hypothesis that patients in investigational antidepressant programs after 2000 had a lowered rate of death by suicide and suicide attempts.

Specifically in the antidepressant group, the number of suicide deaths per 100,000 patient exposure years after 2000 has dropped by 96% of the rate pre-2000 indicating a major shift in the prevalence of depression trial suicide deaths in the past decade and a half. Mirroring this decline, the rate of suicide attempts per 100,000 PEY has also decreased by 84% in programs after 2000. While the decline over time in the suicide death rate of placebo-treated patients was not statistically significant, the significant decrease in the rate of suicide attempts in the placebo group mirrored that of the antidepressant group (an 85% decrease). These decreases were verified with significant findings from regression analyses weighted by program PEY.

These observations are intriguing since there has been a dramatic increase in suicide rates in the general US population in the years following 2000 (Curtin et al. 2016). There are several possible explanations for this decrease in suicide and suicide attempt rates in antidepressant clinical trials in the face of a rising suicide rate in the general population. The first possible explanation that we explored was the potential for enhanced treatment efficacy for suicide prevention among more recent antidepressants. However, support for this theory was not found. The decrease in suicide and suicide attempt rates observed in the antidepressant treatment group were also seen in the placebo group, indicating that medication treatment effects are not likely responsible for the overall observed decline. Additionally, our exploration of drug-placebo differences in suicide and suicide attempt rates revealed no significant differences. We would expect that if medication effects were at play in these data, they would occur exclusively in the antidepressant-treated patients and therefore we would assume that drug-placebo differences in suicide and suicide attempt rates might be more readily observed.

Another potential explanation that we explored was the possibility that key demographic features, such as age and sex, of the patient populations may have changed between pre- and post-2000 programs. In our analysis of these 14 antidepressant programs, we found that the mean age stayed within the range of 40–50 years and did not vary significantly between pre- and post-2000 programs. With regard to percent female, programs consistently enrolled around 60% female patients, with this proportion staying consistent over time. Due to a lack of reporting, analysis of other details of patient demographics (including race, diagnostic history, and familial mental health history) was not possible with these data. However, age and sex are well-established modifiers of suicidality and our findings indicate that these factors have not differentially influenced the suicide and suicide attempt rates between pre- and post-2000 programs.

Given these findings, it is not likely that this phenomenon of decreasing suicide and suicide attempt rates in antidepressant clinical trials reflects changes in antidepressant efficacy or in the demographics of the population sample. A final possible explanation that we considered involves the changes that have occurred in the design and conduct of depression trials. It is possible that this notable decrease in deaths by suicide in more recent antidepressant clinical trials may be partially due to implementation of stringent entry criteria for participation in these trials, including higher awareness of suicide risk by clinical investigators screening patients and pharmaceutical companies’ physicians and scientists. This may additionally be due in part to the increased use of operationalized and systematic suicide risk assessment tools such as the Columbia-Suicide Severity Rating Scale (C-SSRS) (Posner et al. 2011) and the Sheehan Suicidality Tracking Scale (S-STS) (Coric et al. 2009). Due to the proprietary nature of protocol details, reporting of screening procedures is limited within these data and therefore this explanation remains an untested theory. Additionally, selection biases resulting in the exclusion of more severe depression presentations (such as mixed depression or depression with psychotic features that have established increased risk of suicide) may have further suppressed suicidal patients from enrolling in trials of antidepressants.

If screening procedures have become more effective at excluding suicidal patients from clinical trials of antidepressants, then there may be an undesired result—the effects of antidepressant treatment on depressed patients with moderate or high suicidality may not have been able to be evaluated in more recent trials because these patients may have been systematically excluded. On the other hand, the ability to exclude patients with moderate or high suicidality may have a benefit in the reverse. Such effective suicidality screening procedures could be used to intentionally include patients with higher risk for suicidality. Used in this way, clinical trials treating populations at higher risk for suicidality could evaluate the short-term efficacy and the long-term prophylaxis of a candidate anti-suicidality medication. Inclusion of acutely suicidal patients in trials designed to evaluate antidepressant efficacy for suicidality reduction has been successfully modeled in a trial of citalopram combined with lithium (Khan et al. 2011).

There are significant statistical limitations of these data primarily because suicides are rare events and therefore statistical analysis on their occurrence can be severely limited by the amount of exposure. In these data, the deaths by suicide in the placebo group occurred very infrequently overall and PEY for placebo exposure was less than 15% of what it was for antidepressant exposure (see Table 1). Floor effects (in that the number of suicides cannot drop below zero) and reduced statistical power from such inadequate exposure in the placebo group may have limited the ability to find statistical significance for the decrease in the number of deaths by suicide. Because suicide attempts are much more frequent, this measure may have a better sensitivity for detecting changes in the suicidality of patients (both antidepressant and placebo treatment groups showed significant findings in suicide attempts over time).

It is important to note that these data are limited to approved antidepressants only and while suicide data from trials of antidepressants that never gained approval may exist, such data are not publicly accessible and therefore were not available to us for this analysis. Additionally, suicide data may be influenced by the duration of a study—for example in longer term studies, survivor bias may be at play. However, in the case of rare events such as suicide, more events are more likely to be observed over a longer follow-up. Conversely, shorter duration of follow-up may underestimate the number of suicide events. We would have liked to examine any possible effects of the duration of the studies included in the ISS reports; however, the respective contribution of short-term and long-term studies is not given and cannot be accurately estimated from these summary data. Because the placebo data comes primarily from acute, Phase III trials and placebo exposure is generally kept to a minimum, the average follow-up per patient is certain to be less than the antidepressant treatment group which can include open-label, long-term follow-up studies and additional exposure from acute active-controlled trials. Differences between study designs regarding randomization ratios and other pertinent trial design factors also could not be examined because these summary data were pooled. Although we would have liked to use the Poisson regression as a statistical method to examine the continuous decline in suicide rates over time, we were unable to due to the fact that we did not have access to patient-level data. The statistical limitations of rare events and low exposure, particularly in the placebo-exposed group, must be kept in mind when examining these data, but we do not believe that such limitations preclude examining these data with the methods that are available to us.

Finally, because we only have access to data in the public domain (the ISS reports as presented via the Freedom of Information Act on the FDA Access Data website), we are only able to accurately examine suicidal behaviors that are recorded and reported consistently over time. While completed deaths by suicide and suicide attempts have always been considered by the FDA to be adverse events, which are required to be reported in the safety summaries, other measures of suicidality and suicidal behavior have not been consistently measured and reported in these trials. In this context, it is important to note that while measures of suicidality (such as MADRS item-10 or the C-SSRS) have been frequently used in recent years in the selection of patients for trials of antidepressants, these measures are not presented independently as outcome variables in the New Drug Approval packets on the FDA website. Similarly, completed suicides and suicide attempts as this study examined are not presented as outcome measures in the review of efficacy either alternatively or in addition to being reported as adverse events in the safety summary.

What can be concluded from these data is that suicide rates in antidepressant clinical trials are not static—changes in design and procedures have the potential to dramatically alter the rates of suicide and suicide attempts observed within the context of these trials. For this reason, caution must be exercised in extrapolating suicide data from antidepressant trials and generalizing these rates to the greater population.

In conclusion, deaths by suicide and suicide attempts have decreased significantly in antidepressant clinical trials following 2000 compared to the decade before 2000. Basic demographic features of the patients have remained consistent and medication treatment effects on suicidality were not apparent in these data. These findings may reflect enhanced screening procedures and the effective exclusion of suicidal patients in clinical trials for depression.
